# Impact of a warm anomaly in the Pacific Arctic region derived from time-series export fluxes

**DOI:** 10.1371/journal.pone.0255837

**Published:** 2021-08-16

**Authors:** Catherine Lalande, Jacqueline M. Grebmeier, Andrew M. P. McDonnell, Russell R. Hopcroft, Stephanie O’Daly, Seth L. Danielson

**Affiliations:** 1 Amundsen Science, Québec City, Canada; 2 UMCES, Cambridge, Maryland, United States of America; 3 College of Fisheries and Ocean Sciences, University of Alaska Fairbanks, Fairbanks, Alaska, United States of America; University of Palermo: Universita degli Studi di Palermo, ITALY

## Abstract

Unusually warm conditions recently observed in the Pacific Arctic region included a dramatic loss of sea ice cover and an enhanced inflow of warmer Pacific-derived waters. Moored sediment traps deployed at three biological hotspots of the Distributed Biological Observatory (DBO) during this anomalously warm period collected sinking particles nearly continuously from June 2017 to July 2019 in the northern Bering Sea (DBO2) and in the southern Chukchi Sea (DBO3), and from August 2018 to July 2019 in the northern Chukchi Sea (DBO4). Fluxes of living algal cells, chlorophyll *a* (chl *a*), total particulate matter (TPM), particulate organic carbon (POC), and zooplankton fecal pellets, along with zooplankton and meroplankton collected in the traps, were used to evaluate spatial and temporal variations in the development and composition of the phytoplankton and zooplankton communities in relation to sea ice cover and water temperature. The unprecedented sea ice loss of 2018 in the northern Bering Sea led to the export of a large bloom dominated by the exclusively pelagic diatoms *Chaetoceros* spp. at DBO2. Despite this intense bloom, early sea ice breakup resulted in shorter periods of enhanced chl *a* and diatom fluxes at all DBO sites, suggesting a weaker biological pump under reduced ice cover in the Pacific Arctic region, while the coincident increase or decrease in TPM and POC fluxes likely reflected variations in resuspension events. Meanwhile, the highest transport of warm Pacific waters during 2017–2018 led to a dominance of the small copepods *Pseudocalanus* at all sites. Whereas the export of ice-associated diatoms during 2019 suggested a return to more typical conditions in the northern Bering Sea, the impact on copepods persisted under the continuously enhanced transport of warm Pacific waters. Regardless, the biological pump remained strong on the shallow Pacific Arctic shelves.

## Introduction

The Pacific Arctic marine ecosystem is extremely productive due to the persistent flow of nutrient-rich waters fueling high primary production on the shallow northern Bering Sea and Chukchi Sea shelves [1]. Recently, the region showed signs of a warming trend, including drastic reductions in sea ice extent and an increase in the transport of warm Pacific waters delivering more heat and freshwater, and potentially more nutrients and biota, into the Arctic Ocean through the Bering Strait [1–8]. Amid this long-term warming trend, anomalously warm conditions were observed in the Pacific Arctic from 2017 into 2019, including an unprecedented loss of sea ice, even in the context of other recent warm years [1, 9, 10]. Sea ice cover barely extended south of Bering Strait in early January 2017 and remained well below the long-term average during the entire winter [1, 11]. The reduced sea ice cover enhanced oceanic heat uptake during spring months [6] and led to exceptionally high near-bottom ocean temperatures (~4°C) in the Bering Strait in June 2017 [1]. The anomalously elevated water column heat content combined with winds from the south delayed sea ice formation to late December 2017 and contributed to the highest northward transport of Pacific waters during winter 2017–2018 [1, 6, 12]. Warm winds from the south also contributed to the record-breaking low sea ice extent and concentrations observed in the Pacific Arctic region in February 2018 and to the northern Bering Sea region being mostly ice-free by late March 2018 [1, 13–15]. The early sea ice retreat and reduced input of freshwater from melting sea ice in 2018 delayed the onset of stratification and the spring bloom [14, 16–18], and induced a shift in the composition of the phytoplankton community toward a high abundance of small diatoms [14]. Meanwhile, low abundance of large, lipid-rich copepods and high abundance of small copepods with low lipid content were reported in the northern Bering Sea during 2018 [1, 14–16], affecting the distribution of fish as well as the reproduction and survival of marine birds and mammals [14]. Nevertheless, flux measurements obtained using sediment traps deployed in the Bering Strait region during June 2018 highlighted remarkably high POC fluxes during the warm period [19]. Similar to 2017, accumulated residual heat reflected by record high sea surface temperatures during autumn 2018 [20] delayed freeze-up until December, while unusual southerly winds again forced a large ice retreat in February 2019 that led to the second lowest winter sea ice extent on record during 2018–2019 [1, 13].

**Fig 1:**
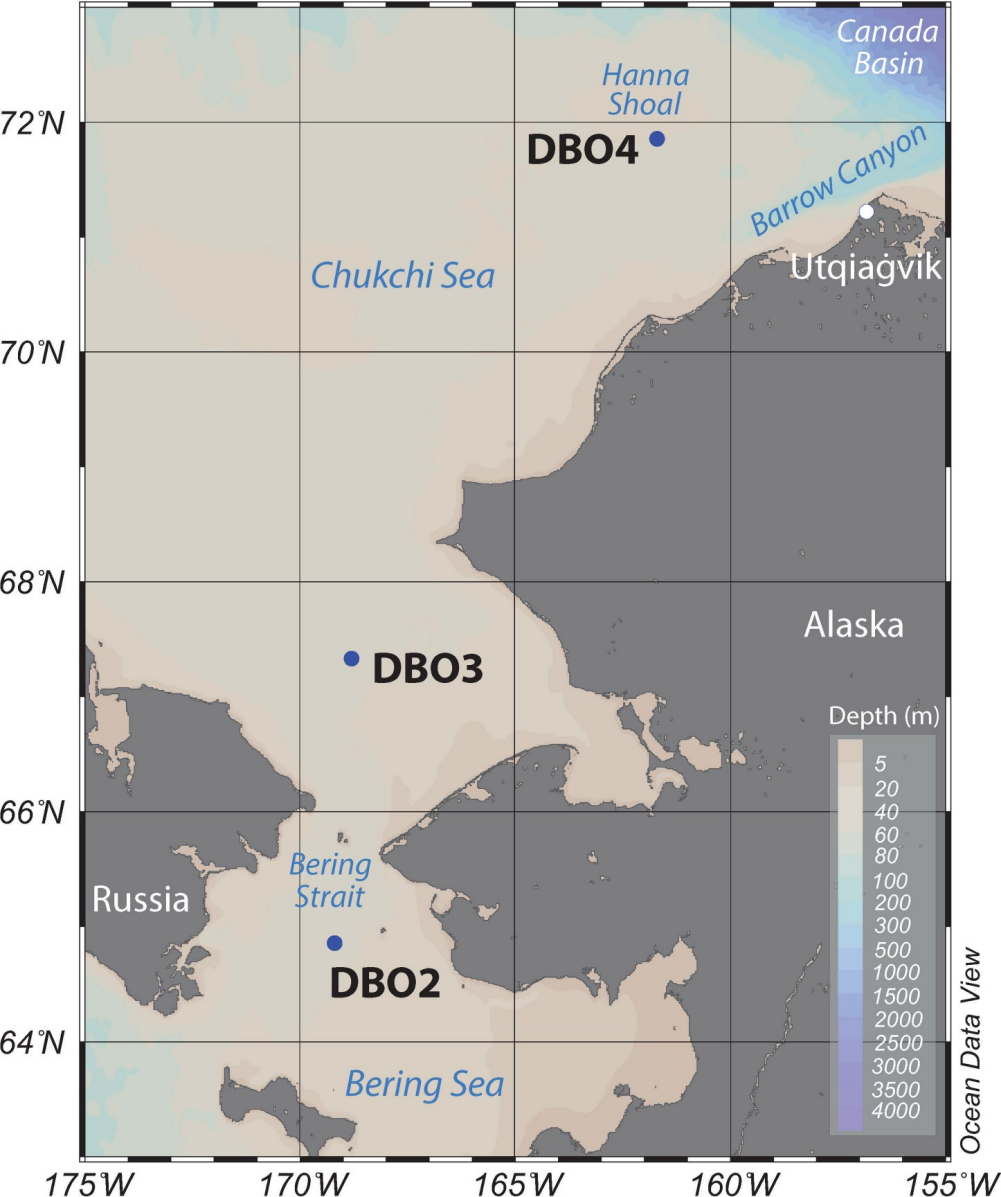
Positions of the three mooring sites at the DBO hotspots in the Pacific Arctic region. Reprinted from Ocean Data View under a CC BY license, with permission from Reiner Schlitzer, original copyright 2021.

While results from several snapshot studies clearly indicated that a sudden shift of the Pacific Arctic ecosystem occurred during the anomalously warm 2017–2019 period, most biological measurements were limited to summer months [1]. To complement these snapshot observations, sinking particles and plankton collected with moored sediment traps deployed at three sites of the Distributed Biological Observatory (Grebmeier et al., this issue) were used to monitor spatial and temporal variations in export fluxes and community composition in the Pacific Arctic region. Continuous flux measurements were obtained at the DBO2 and DBO3 sites from June 2017 to July 2019 and at the DBO4 site from August 2018 to July 2019 (Fig 1). This monitoring effort using sequential sediment traps follows the first time-series flux measurements obtained on the shallow Pacific Arctic shelves at the Chukchi Ecosystem Observatory (DBO4) from August 2015 to July 2016 [21]. Fluxes of living algal cells (with chloroplasts), chlorophyll *a* (chl *a*), total particulate matter (TPM), particulate organic carbon (POC), and zooplankton fecal pellets were measured to monitor carbon export and the seasonal development of the algal bloom in relation to the drastic changes observed during the warm anomaly period. Zooplankton and meroplankton collected in the sediment traps were also identified to track spatial and temporal variations in the composition and development of these communities. The deployment of moored sediment traps represents an invaluable contribution to the DBO as they provided time-series integrative measurements of biological and biochemical parameters at a high temporal resolution during a period of rapid changes in the Pacific Arctic. As warm events are likely to increase in frequency under the current global warming scenario, time-series export fluxes measurements obtained during the 2017–2019 warm period contribute to a better understanding of conditions to expect for the highly productive marine ecosystems of the northern Bering Sea and Chukchi Sea.

## Material and methods

### Remote sensing

Daily averaged sea-ice concentrations above each mooring site were retrieved at a 12.5-km resolution from the Centre ERS d’Archivage et de Traitement (CERSAT) service of the French Research Institute for Exploitation of the Sea (http://cersat.ifremer.fr/). Daily sea-ice concentrations were averaged for a 44 x 44 km region above each mooring site (Fig 1; DBO2: 64.7–65.1°N; 169.3–169.8°W; DBO3: 67.1–67.5°N; 168.5–169.0°W; DBO4: 71.4–71.8°N; 161.4–161.9°W).

### Mooring

Sequential sediment traps (24 cups, Hydro-Bios, Germany) were deployed at the DBO2 (N4) and DBO3 (N6) sites from June 2017 to July 2019 and at the DBO4 (Chukchi Ecosystem Observatory; CEO) site from August 2018 to July 2019 (Fig 1 and Table 1). No national or international permitting was required as part of the sample collection efforts, and the field studies did not involve endangered or protected species. At CEO, year-round measurements capture physical and biogeochemical parameters in addition to measures of fish, zooplankton, marine mammals, and other ecosystem components [e.g. 22–24]. Moorings were deployed and recovered from the R/V *Sikuliaq* in June 2017 and June 2018 and recovered from the R/V *Ocean Starr* in July 2019. Sediment trap sample cups were filled with filtered seawater adjusted to a salinity of 38 with NaCl and fixed with formalin (4% final solution) to preserve samples during deployment and after recovery. The carousel holding the sample cups rotated at pre-programmed intervals ranging from one week to one month. As the mooring was recovered before the last rotation of the carousel at DBO2 in June 2018, the sample cup that was open upon recovery was excluded from analysis. Seabird SBE 16plus units were deployed to measure water temperature at three depths at DBO2 and DBO3 and at one depth at DBO4. The unit deployed at the upper depth on each mooring was coupled with a WETStar fluorometer or WET Labs ECO triplet fluorometer to measure fluorescence (Table 1).

**Table 1:** Mooring and sediment trap deployment information.

Site	Mooring name	Latitude (N)	Longitude (W)	Sampling start date	Sampling end date	Water depth (m)	Sediment trap depth (m)	Fluorometer depth (m)	CTD depth (m)
DBO4	CEO	71˚35	161˚31	August 7 2018	July 30 2019	46	39	33	33
DBO3	N6	67˚40	168˚44	June 17 2017	June 8 2018	50	35	26	26, 35, 45
				June 16 2018	July 13 2019	50	35	25	26, 46
DBO2	N4	64˚55	169˚55	June 26 2017	June 8 2018	49	37	27	27, 35, 44
				June 24 2018	July 13 2019	49	37	25	25, 35, 44

### Laboratory

In the laboratory, zooplankton and meroplankton were removed from subsamples with forceps and identified to the lowest taxonomic level possible using a dissecting microscope. Sample cups were gently mixed before subsamples (0.1–3 ml) were taken with a modified micropipette to enable the collection of large particles for measurements of chl *a*, algal cells, fecal pellets, TPM, and POC. Subsamples for chl *a* measurements were filtered onto GF/F filters (0.7 μm), extracted in acetone for 24 h at -20°C and measured on a Turner Design fluorometer following the methods outlined in Welschmeyer [25]. Samples were kept cool and in the dark prior to chl *a* measurements, but may have experienced some degradation. For the enumeration of algal cells, subsample volumes were adjusted to 3 ml with filtered seawater before being placed in an Utermöhl chamber. A minimum of 300 algal cells were counted and identified by inverted microscopy at 100X, 200X or 400X depending on cell size according to the Utermöhl method [26]. Using a dissecting scope, the length and width of fecal pellets (broken or intact) were measured with an ocular micrometer and fecal pellet volumes were calculated according to their shape. Cylindrical pellets were attributed to calanoid copepods while ellipsoidal pellets were attributed to appendicularians [27]. Fecal pellet volumes were converted to fecal pellet carbon (FPC) using a volumetric carbon conversion factor of 0.057 mg C mm^-3^ for copepod pellets and 0.042 mg C mm^-3^ for appendicularian pellets [27]. Subsamples for TPM measurements were filtered in triplicate onto pre-combusted (500°C overnight) and pre-weighed GF/F filters (0.7 μm), rinsed with distilled water to remove salt, dried at 60°C overnight, and weighed on a microbalance. The filters were then exposed to 1N HCl overnight for removal of inorganic carbon and dried once again at 60°C overnight before encapsulation for POC measurements. POC was measured using a PerkinElmer CHNS 2400 Series II elemental analyzer. All measurements were converted to daily fluxes depending on sampling duration and integrated to annual fluxes.

## Results

### Sea ice concentrations

**Fig 2:**
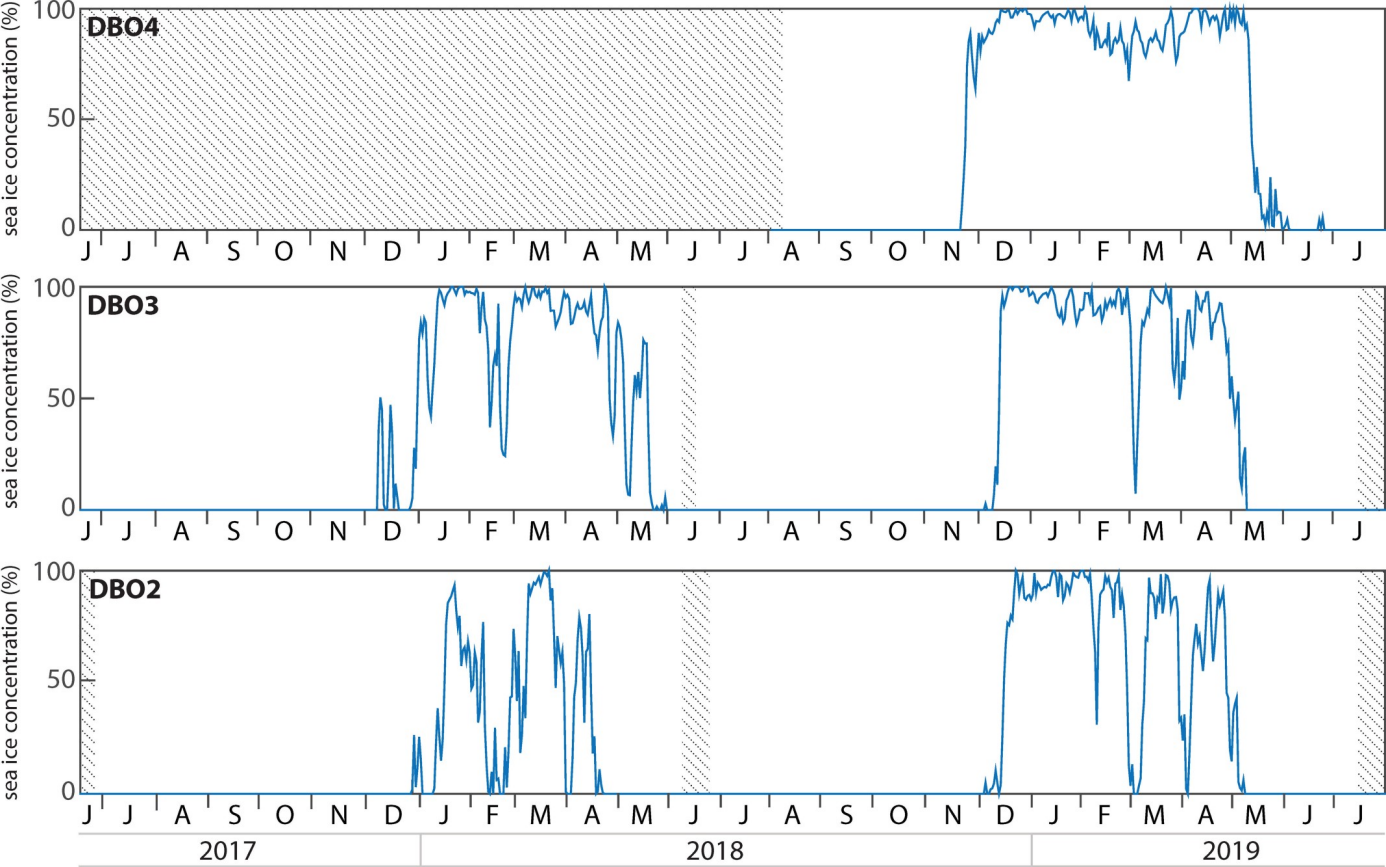
Daily sea ice concentration retrieved for a delimited region above each mooring site. Shaded areas represent periods without data.

While sea ice was first detected in December 2017 at DBO2 and DBO3, a lasting sea ice cover only formed in January 2018 and sea ice concentrations temporarily decreased in February 2018 at both sites (Fig 2). At DBO2, sea ice concentrations decreased again in early April before complete sea ice melt was observed at the end of April 2018. Sea ice melt was observed one month later at the end of May 2018 at DBO3. Sea ice formed again in early December 2018 at DBO2 and DBO3, two to three weeks earlier than when sea ice formed during 2017. Sea ice concentrations frequently decreased throughout winter 2018–2019, especially at DBO2. At DBO4, sea ice formed in November 2018, two to three weeks earlier than at the other sites, and sea ice concentrations remained >65% throughout winter. Sea ice breakup was observed in early May 2019 at DBO2, a few days later at DBO3, and mid-May at DBO4. While sea ice melted approximately three weeks later in 2019 than in 2018 at DBO2, it disappeared approximately two weeks earlier in 2019 than in 2018 at DBO3.

### Water temperatures

**Fig 3:**
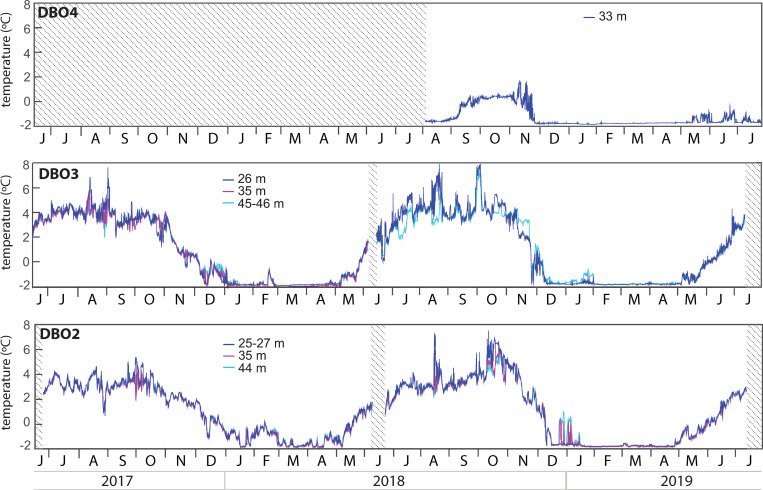
Water temperatures at each mooring site. There are no available data at 35 m for the 2018–2019 cycle at DBO3. Shaded areas represent periods without data.

Mooring-derived water temperatures were above 2°C from June to November at all depths sampled at DBO2 and DBO3 (Fig 3). Water temperatures frequently peaked between 6°C and 8°C at both sites during late summer and autumn, except at DBO2 in 2017 where they remained below 6°C. At DBO2, water temperatures slowly decreased to near-freezing point (-1.8°C) only during very short periods of January, March and April 2018, while in 2019, they decreased more rapidly and remained near the freezing point from January until the end of April. At DBO3, water temperatures were almost continuously near the freezing point from January to early May 2018 and from December 2018 to early May 2019. At DBO4, water temperatures exceeded 0°C but remained below 2°C from September to November 2018 and were near the freezing point from the end of November 2018 to May 2019.

### Chlorophyll a concentrations and fluxes

**Fig 4:**
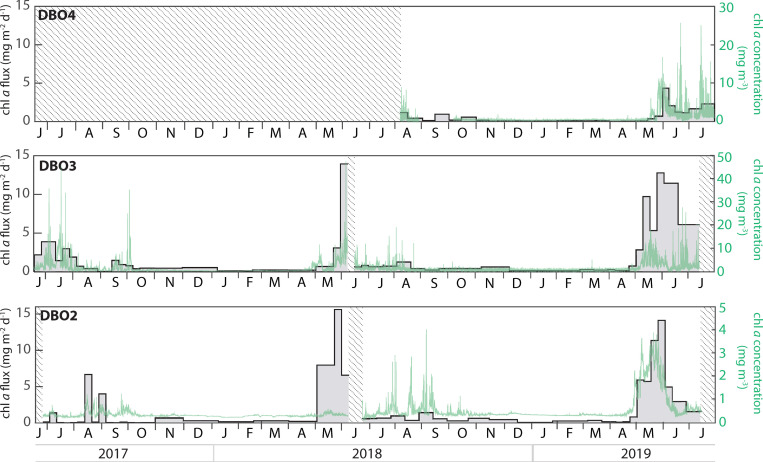
Chlorophyll *a* fluxes (grey bars) and suspended chlorophyll *a* concentration (green line) at each mooring site. Shaded areas represent periods without data.

While enhanced chl *a* fluxes often coincided with increased suspended chl *a* concentrations recorded by the fluorometer 6 to 10 m above the traps, there were also notable differences between the two measurements (Fig 4). Peak chl *a* fluxes were observed at the end of May and/or early June for every deployment cycle, reaching values >10 mg m^-2^ d^-1^ at DBO2 and DBO3. During 2017, short periods of enhanced chl *a* fluxes were observed in August at DBO2 and during June and July at DBO3. Although there was a period without sampling due to the timing of the mooring turnaround in 2018, elevated chl *a* fluxes (>5 mg m^-2^ d^-1^) were sustained during approximately five weeks in 2018 and 2019 at DBO2, and during two weeks in 2018 and nine weeks in 2019 at DBO3. At DBO4, relatively low chl *a* fluxes peaked in early June 2019 (4.3 mg m^-2^ d^-1^) and remained above >1 mg m^-2^ d^-1^ until the end of the deployment in late July 2019.

### Diatom fluxes

**Fig 5:**
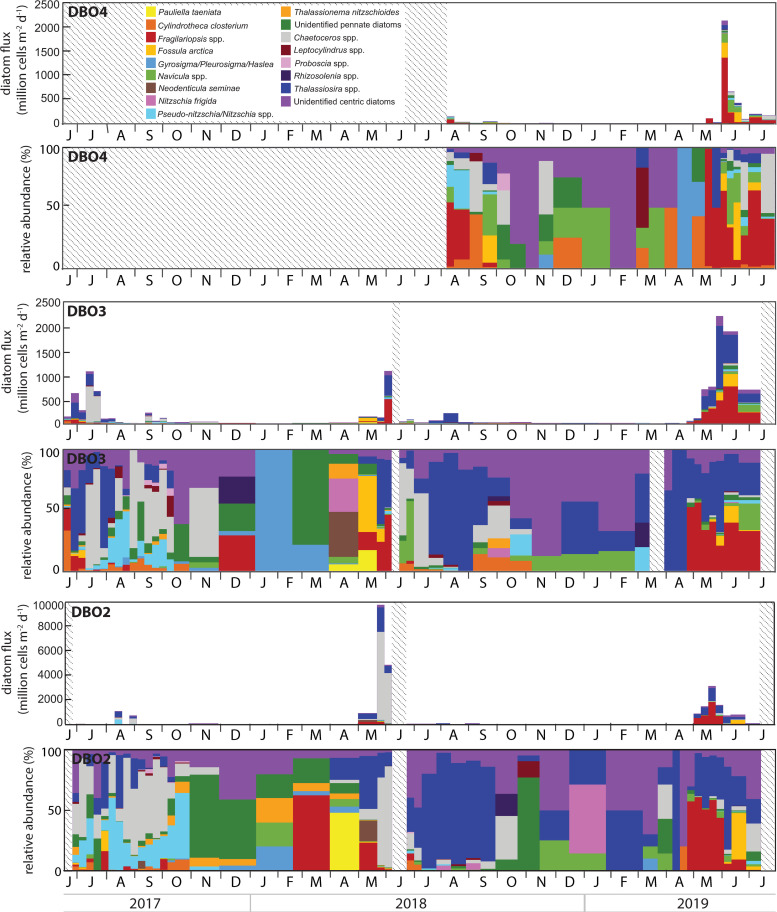
Diatom fluxes and relative abundance of the dominant diatom groups at each mooring site. Note the different scales. Shaded areas represent periods without data.

Temporal variations in fluxes of diatom cells with chloroplasts were remarkably similar to chl *a* fluxes, with peak diatom fluxes observed at the end of May and/or early June for every deployment cycle (Fig 5). Whereas diatom fluxes were higher at DBO3 than DBO2 during summer 2017, the composition of the fluxes was similar at both sites, with the centric diatoms *Chaetoceros* spp. and *Thalassiosira* spp. and the pennate diatoms *Pseudo-nitzschia*/*Nitzschia* spp. contributing a large proportion of the fluxes. Low fluxes of the pennate diatom *Thalassionema nitzschioides* and the centric diatoms *Leptocylindrus* spp. and *Proboscia* spp. were also frequently observed during summer and autumn 2017. At DBO2, enhanced diatom fluxes with large proportions of the pennate diatoms *Fragilariopsis* spp. and *Neodenticula seminae* in early May 2018 were followed by extremely large fluxes nearly exclusively composed of *Chaetoceros* spp. and *Thalassiosira* spp. during late May and early June 2018. At DBO3, enhanced fluxes during May 2018 were first composed of the pennate diatoms *Pauliella taeniata* and *Fossula arctica* before *Fragilariopsis* spp. and *Thalassiosira* spp. dominated the peak in diatom export in early June 2018. Diatom fluxes at both DBO2 and DBO3 were much lower after mooring turnaround during June 2018, gradually shifting to diatom fluxes dominated by *Thalassiosira* spp. during summer 2018. At DBO4, low diatom fluxes during summer 2018 were mostly composed of *Fragilariopsis* spp., *Pseudo-nitzschia*/*Nitzschia* spp., *Cylindrotheca closterium*, and *Chaetoceros* spp. Diatom fluxes increased again during May 2019 at DBO2 and DBO3 and in early June 2019 at DBO4. The magnitude and composition of the fluxes during spring 2019 was very similar at the three DBO sites, with *Fragilariopsis* spp., *Thalassiosira* spp., *Fossula arctica*, *Navicula* spp., *Pseudo-nitzschia*/*Nitzschia* spp., and *Chaetoceros* spp. contributing to the elevated fluxes.

### Zooplankton and meroplankton

**Fig 6:**
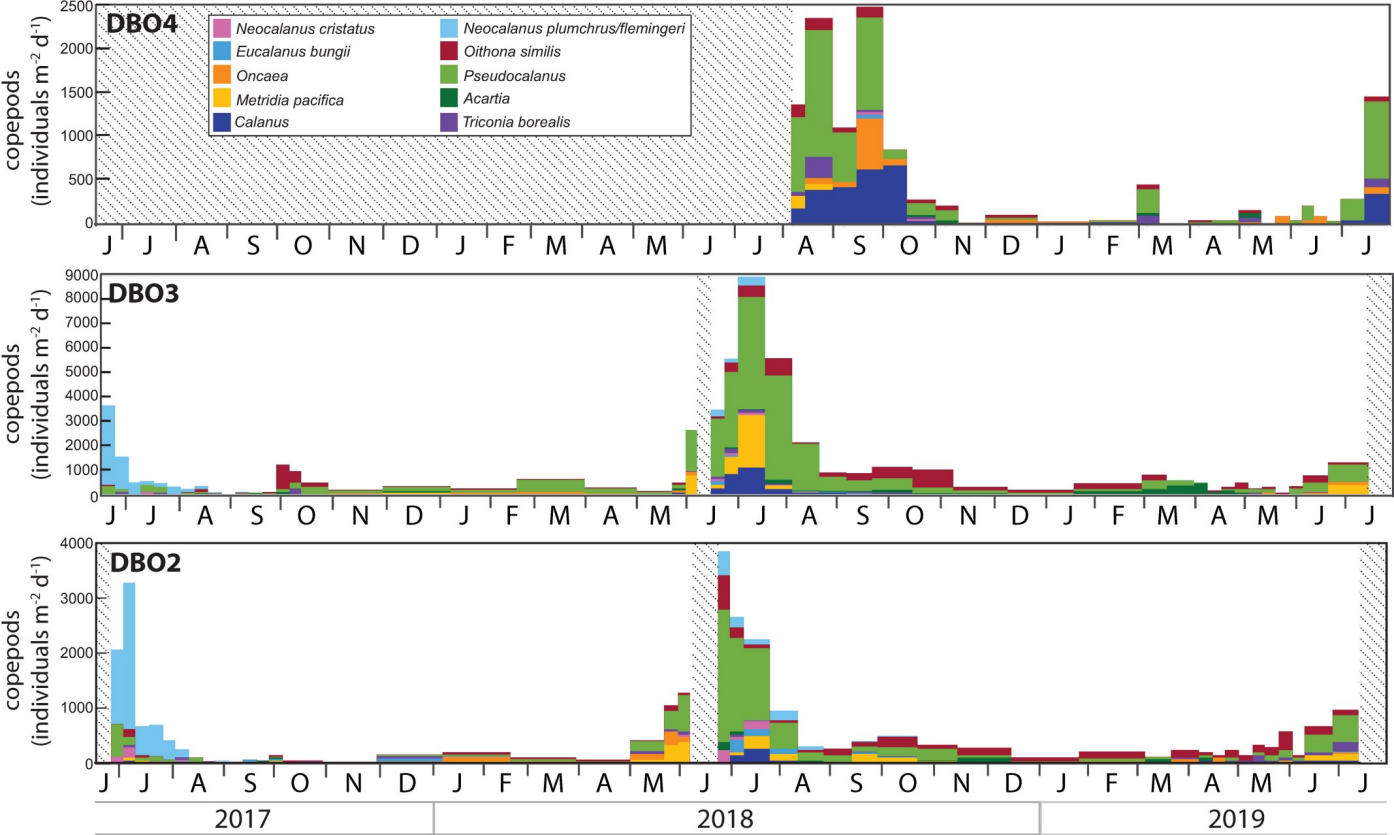
Dominant copepod groups collected in the sediment trap at each mooring site. Note the different scales. Shaded areas represent periods without data.

**Fig 7:**
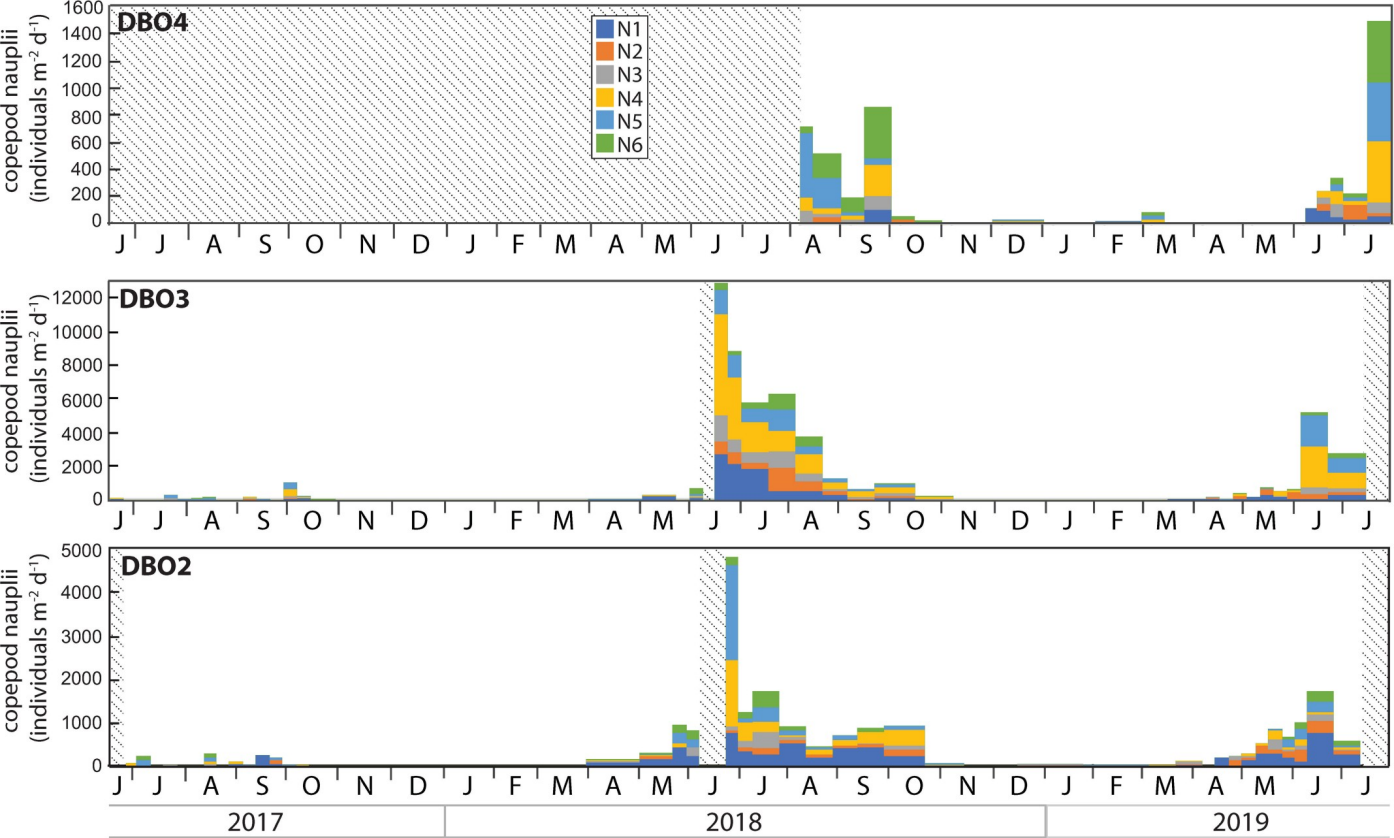
Nauplii of the copepod genus *Calanus*, *Metridia* and *Pseudocalanus* collected in the sediment trap at each mooring site. Note the different scales. Shaded areas represent periods without data.

**Fig 8:**
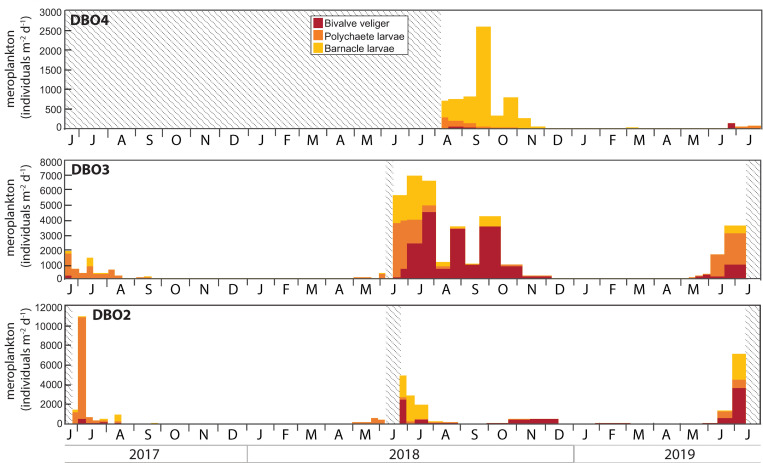
Meroplankton larvae collected in the sediment trap at each mooring site. Note the different scales. Shaded areas represent periods without data.

Copepods, copepod nauplii, and meroplankton were the most numerous animals collected in the sediment traps, with the highest abundances observed during June or July at DBO2 and DBO3 and during late September at DBO4 (Figs 6–8). As morphological separation of all life stages of several dominant copepod species in the Pacific Arctic is difficult, most species were aggregated and reported at the generic level (e.g. *Calanus glacialis*, *C*. *marshallae*; *Neocalanus plumchrus*, *N*. *flemingeri*; *Pseudocalanus minutus*, *P*. *acuspes*, *P*. *mimus*, and *P*. *newmani*), with the exception of *N*. *cristatus* that is recognizable due to its large size compared to its congeners [28]. A distinct dominance of the Pacific copepods *Neocalanus* was observed at DBO2 and DBO3 from June to August 2017 (Fig 6). The composition of the copepod community shifted to a dominance of *Pseudocalanus* at all sites during spring and/or summer 2018, with varying abundances of *Metridia*, *Calanus*, and *Oncaea* among sites.

Whereas few nauplii of the genera *Calanus*, *Metridia*, and *Pseudocalanus* were collected during summer 2017, large numbers of them were collected during summer 2018 and 2019, especially at DBO3 (Fig 7). At DBO2 and DBO3, nauplii of all stages were often collected simultaneously from May to October, with a larger proportion of nauplii stages N1 and N2 collected during April and May and peaks in nauplii abundance recorded in June. At DBO4, a large proportion of stages N5 and N6 was recorded during August and September 2018. Nauplii of all stages were again collected during June 2019 and peaked in abundance at the end of July 2019.

Meroplanktonic stages were dominated by polychaete larvae during summer 2017 at DBO2 and DBO3, while high abundances of bivalve veliger, polychaete larvae, and barnacle larvae blended from spring to autumn 2018 and during spring and early summer 2019 (Fig 8). At DBO4, barnacle larvae contributed to the vast majority of meroplankton collected from August to November 2018, but were absent during June and July 2019 when few bivalve veliger and polychaete larvae were collected.

### Particulate organic carbon and total particulate matter fluxes

**Fig 9:**
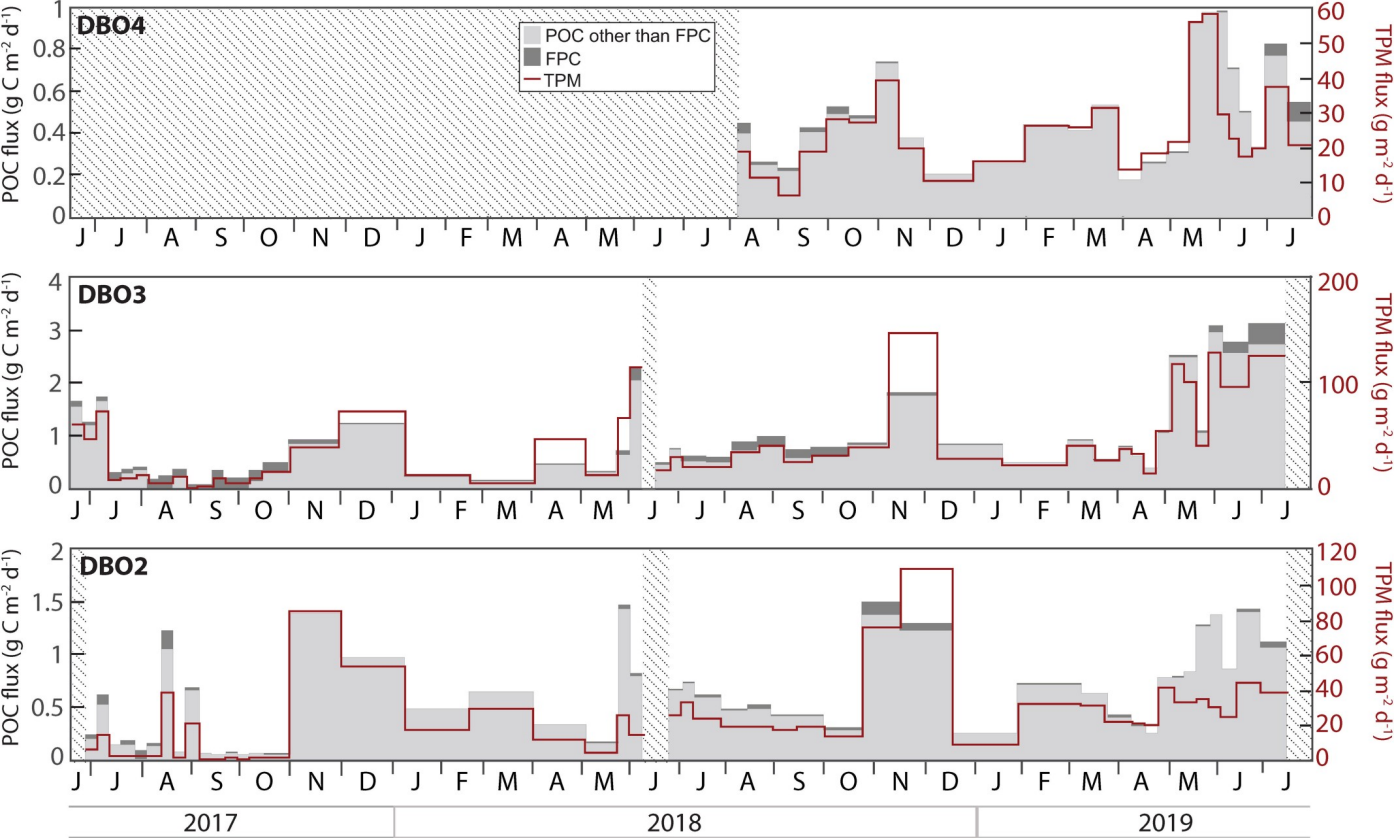
Particulate organic carbon (POC), fecal pellet carbon (FPC), and total particulate matter (TPM) fluxes at each mooring site. Note the different scales. Shaded areas represent periods without data.

POC and TPM fluxes displayed similar temporal variations at DBO2 and DBO3 (Fig 9). At DBO2, POC and TPM fluxes increased simultaneously except during spring when TPM fluxes remained relatively low. At DBO3, the highest POC fluxes of the Pacific Arctic region (>2 g m^-2^ d^-1^) were observed in early June 2018 and from May to July 2019, except for a week at the end of May 2019. At DBO4, peak POC (~1 g m^-2^ d^-1^) and TPM fluxes (>50 g m^-2^ d^-1^) were recorded during the second half of May 2019. High POC and TPM fluxes were also observed during autumn and winter at all sites, often in November and/or December. FPC fluxes contributed most to POC fluxes between June and November at all sites, and sporadically contributed to the complete POC flux between August and October 2017 at DBO3 (Fig 9).

### Annual fluxes

**Fig 10:**
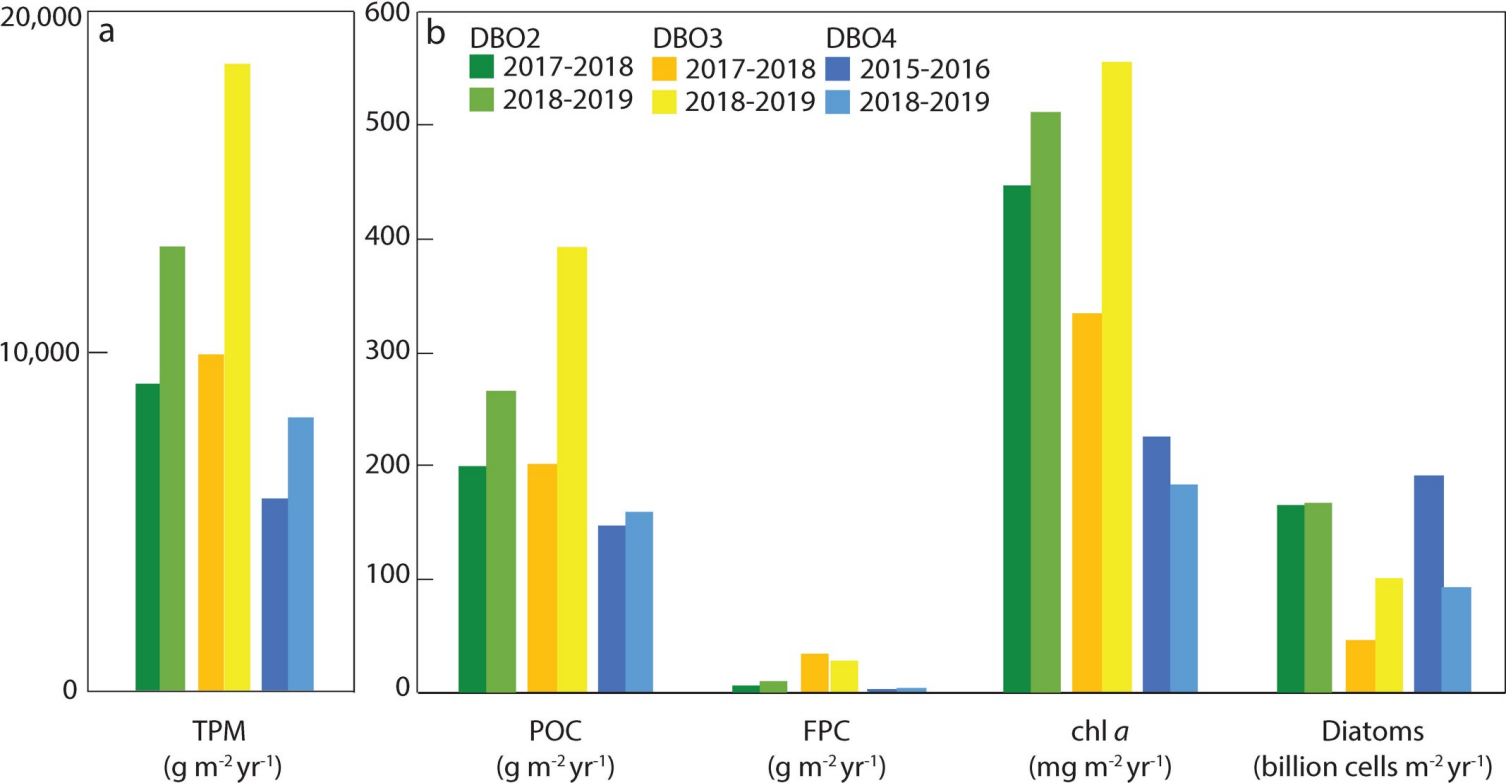
a) Annual total particulate matter (TPM) fluxes, and b) annual particulate organic carbon (POC), fecal pellet carbon (FPC), chlorophyll *a* (chl *a*), and living diatom fluxes at the three mooring sites in the Pacific Arctic region.

Annual fluxes of TPM, POC, FPC, chl *a*, and diatoms all increased during the 2018–2019 cycle at DBO2 (Fig 10). Annual fluxes were also higher during the 2018–2019 cycle than during the 2017–2018 cycle at DBO3, except for a lower annual FPC flux. At DBO4, annual TPM, POC, and FPC fluxes obtained from August 2018 to July 2019 were higher than previously measured under extended sea ice cover at the same site from August 2015 to July 2016 [21], while annual chl *a* and diatom fluxes were lower during 2018–2019 than during 2015–2016 [21].

## Discussion

A combination of downward export, lateral advection, and resuspension of particles contributed to the fluxes recorded at the three mooring sites of the shallow Pacific Arctic region. As typically observed on Arctic shelves [29, 30], TPM and POC fluxes were strongly correlated (R^2^ = 0.82, p<0.01) at the DBO sites, with POC fluxes consistently contributing to <7% of the TPM fluxes. Whereas enhanced fluxes in April or May most likely resulted from the release of particulate matter from the melting ice [21], algal export contributed to the elevated POC fluxes during May and June. Diatoms usually dominate phytoplankton abundance and biomass in the northern Bering Sea and Chukchi Sea [31–34] and typically represent the most important component of algal fluxes across the Arctic Ocean due to their rapid sinking velocities [21, 30, 35, 36]. Diatoms also dominated algal export during spring at the three DBO sites, contributing to daily POC fluxes that were among the highest ever documented in the global oceans [19].

Zooplankton continuously collected at fixed depths indicated a strikingly similar composition of the dominant copepods at the three DBO sites, consistent with the large influence of advection on seasonal zooplankton dynamics in the Pacific Arctic [37, 38]. Copepod grazing certainly reduced diatom fluxes during summer, as supported by enhanced FPC fluxes. Similar to copepod and copepod nauplii abundances, benthic larvae abundance generally increased after the onset of the algal bloom, with large numbers of bivalve veliger, polychaete larvae, and barnacle larvae contributing to the grazing pressure on diatoms during summer and autumn. The lasting presence of planktonic larvae of benthic animals indicated productive conditions conducive to reproduction during several weeks at all sites. While copepod fecal pellets enhanced POC fluxes during summer and autumn, elevated TPM and POC fluxes during autumn and winter likely resulted from wind-induced resuspension and dispersion of sediments, as current speeds reached velocities >40 cm s^-1^ during autumn 2017 at DBO2 and DBO3 [19]. Short-lived increases in chl *a* concentrations, chl *a* fluxes, and diatom fluxes sporadically observed during late summer and autumn further support wind-induced resuspension and dispersion of sediments containing diatoms and chl *a* at the DBO sites. These observations are in agreement with enhanced near-bottom chl *a* concentrations observed during wind-induced high turbidity events during autumn near Bering Strait [39, 40], and with low fluxes of the sympagic diatom biomarker IP_25_ and of diatoms containing chloroplasts throughout the polar night on the northeast Chukchi shelf [21, 41]. In addition to reflecting the seasonal processes influencing the export of particulate matter in the region, the nearly continuous measurements obtained at a high temporal resolution from June 2017 to July 2019 were used to assess the impact of the enhanced transport of warmer Pacific waters and shorter ice-covered duration on the Pacific Arctic marine ecosystem.

### June 2017—June 2018

An earlier onset of warming and later onset of cooling in recent years lengthened the warm (≥0°C) water duration to more than 6 months in the Pacific Arctic region [7]. In 2017, the warm period lasted over 7 months and mean June temperatures were 2°C warmer than climatology when the sediment traps were deployed [7]. A spring bloom probably occurred prior to the onset of sampling at both sites, as indicated by elevated near-bottom chl *a* concentrations revealing the occurrence of a spring bloom during May 2017 at a nearby mooring site in the southern Chukchi Sea [17, 39]. However, relatively high diatom fluxes mostly composed of the exclusively pelagic diatoms *Chaetoceros* spp. and *Thalassiosira* spp. indicated that a pelagic bloom occurred in the DBO3 region during late June and July 2017. The subsequent frequent collection of the boreal diatoms *Proboscia* spp., cosmopolitan diatom *T*. *nitzschioides*, and endemic North Pacific diatom *Neodenticula seminae* [31, 33, 42, 43] during summer and autumn reflected the enhanced inflow of warm Pacific waters across Bering Strait during 2017.

Although zooplankton community composition and distribution have been shown to vary with water properties and the volume of Pacific water transported to the northern Bering Sea and Chukchi Sea [38, 44–47], the most abundant groups are generally found throughout the region and differences between communities often depend on the presence or absence of indicator species [4]. Accordingly, peaks in copepod abundance dominated by the Pacific copepods *Neocalanus* during late June and early July 2017 highlighted an enhanced transport of warm Pacific waters, in agreement with concurrent observations of high abundance of Pacific copepods associated to the inflow of Anadyr Water during June and July 2017 in the northern Bering Sea [44]. The very low numbers of nauplii collected during 2017 reflected the low abundance of the three copepod groups for which nauplii were identified in the trap samples. In particular, the absence of *Calanus* copepodites and nauplii indicated their decline under warm conditions, as supported by the report of a remarkably low abundance of *Calanus* in the Pacific Arctic during 2017 [1]. The nearly exclusive presence of polychaete larvae during 2017 also suggested their transport with the enhanced inflow of Pacific waters.

Residual heat from the elevated summer water temperatures delayed sea ice formation to December 2017 at both sites [6]. At DBO2, this delay reduced the rejection of salty, cold, and dense brine that typically sinks to the bottom to form a cold water layer, thereby limiting the cooling of the water column during winter and contributing to significant heat input into the Arctic Ocean [13]. At DBO3, oceanic heat loss was sufficient to reduce water column temperature at the freezing point during winter. As oceanic heat flux through Bering Strait triggers the onset of ice melt in the shallow Pacific Arctic region [7], the enhanced heat input during winter presumably contributed to the early ice breakup observed at DBO2 during spring 2018. High chl *a* and diatom fluxes dominated by the exclusively pelagic diatoms *Chaetoceros* spp. and *Thalassiosira* spp. in late May clearly indicated the occurrence of a large open water bloom following the early sea ice breakup in the northern Bering Sea. While biofouling may explain the concurrent low chl *a* concentrations recorded at DBO2 at the end of May, high chl *a* and diatom fluxes at that time were in agreement with the peak near-bottom chl *a* concentrations recorded at a nearby mooring on May 20 2018 [17]. The early collection of low numbers of the ice-associated pennate diatoms *Fragilariopsis* spp., *F*. *arctica*, and *Navicula* spp. during the sea ice breakup period (sea ice concentration <50%) hinted at a limited ice algae production while ice still drifted in the region in April, inhibiting the seeding of the spring bloom [48] and likely contributing to the shift in the composition of the diatom fluxes. Peak diatom fluxes at the end of May 2018 occurred approximately seven weeks after sea ice breakup at DBO2, likely due to weaker stratification following the extremely low winter sea ice extent and concentration, as observed during July 2018 in the northern Bering Sea [18]. Hence, the early sea ice breakup inhibited the ice-edge bloom and resulted in a large open water bloom in the northern Bering Sea, similar to previous observations following early sea ice breakup in the southeastern Bering Sea [17, 49, 50]. At DBO3, the coincident sea ice breakup and export of ice-associated pennate diatoms in early May was followed approximately three weeks later by a peak in chl *a* and diatom fluxes composed of ice-associated and pelagic diatoms during early June, indicating that colder water temperatures, later sea ice melt, and stronger stratification led to an ice-edge bloom in the southern Chukchi Sea in contrast to the northern Bering Sea. The highest near-bottom chl *a* concentrations recorded in late May and early June 2018 at a nearby mooring in the DBO3 region [17] confirmed that most of the bloom occurred before mooring turnaround in 2018. In response to the enhanced diatom fluxes, the abundances of copepods, copepod nauplii, and polychaete larvae increased at both sites during May 2018.

### June 2018—June 2019

Low chl *a* and diatom fluxes mostly composed of *Chaetoceros* spp. and *Thalassiosira* spp. when sampling was resumed during June 2018 at DBO2 and DBO3 indicated a declining pelagic bloom at both sites. From July to September, the centric diatoms *Thalassiosira* spp. dominated the low diatom fluxes, a sharp contrast with fluxes composed of pennate diatoms *Pseudo-nitzschia*/*Nitzschia* spp., *C*. *closterium*, *Fragilariopsis* spp., *F*. *arctica*, and centric diatoms *Chaetoceros* spp. collected during the previous summer. This shift in flux composition concurs with a lower abundance of *Chaetoceros* spp. observed during July 2018 than during July 2017 in the Bering Strait region [49]. The large proportion of *Thalassiosira* spp. in the low diatom fluxes during July 2018 possibly reflected a rapid depletion of nutrients during the large open water bloom taking place in the northern Bering Sea weeks earlier.

Peaks in copepod abundance during late June at DBO2 and early July at DBO3 probably contributed to the low diatom fluxes observed in early summer through grazing pressure. Most of the copepods collected during June and July 2018 consisted of *Pseudocalanus* while the abundance of *Neocalanus* and *Calanus* remained relatively low at both sites. As *Pseudocalanus* copepods are approximately three times smaller than *Neocalanus* and *Calanus*, their dominance supports observations of a zooplankton community dominated by small copepods during 2018 [15]. While small copepods *Pseudocalanus*, *Acartia*, and *Oithona similis* often dominate copepod abundance, large *Calanus* copepods often dominate biomass in late summer in the southern Chukchi Sea [38]. As *C*. *glacialis* females exploit ice algae to fuel their maturation and egg production [51, 52], the abundance and biomass of *C*. *glacialis* usually increase under colder conditions in the Bering and Chukchi Seas [53, 54]. The dominance of small copepods in 2018 therefore resulted from a low abundance of *C*. *glacialis* under warmer conditions. The presence of the Pacific copepods *Neocalanus*, *Eucalanus bungii* and *Metridia pacifica* [32, 44–46] from June to October 2018 further illustrated the influence of warm Pacific waters.

At DBO4, low diatom fluxes composed of *Fragilariopsis* spp., *C*. *closterium*, *Pseudo-nitzschia/Nitzschia* spp., *Navicula* spp., *Thalassiosira* spp., and *Chaetoceros* spp. at the onset of sampling in August 2018 corresponded to the ice-associated and pelagic diatom groups reported to dominate phytoplankton biomass during summer in the northern Chukchi Sea [33, 55–59], albeit with variations in the relative contribution of each group. Despite the absence of measurements prior to August 2018, the distinct composition of diatoms commonly observed during summer near Hanna Shoal suggests that local algal production was not affected by the anomalous 2018 conditions observed south of Bering Strait. Previous diatom flux measurements at the DBO4 site showed very large fluxes of *C*. *closterium* from August to October 2015 during a period of strong winds with frequent direction reversals [21]. Similarly, a strong wind event in September 2013 at a nearby site in the Chukchi Sea induced sufficient vertical mixing to enhance phytoplankton productivity and led to high abundances of *C*. *closterium* and *Leptocylindrus danicus* [60, 61]. However, although high water temperatures recorded at depth during November 2018 indicated storm conditions mixing the water column at DBO4, *C*. *closterium* fluxes remained low during autumn 2018 when sunlight was sufficient to induce primary production in the region. Peaks in copepod abundance recorded during late August and late September 2018 at DBO4 were dominated by small *Pseudocalanus*, similar to DBO2 and DBO3. The dominance of small copepods in the northern Chukchi Sea also resulted from a low abundance of *C*. *glacialis* under warmer conditions, as confirmed by *Calanus* that were ~5 times more abundant at DBO4 during August 2015 than during August 2018 while the abundance of *Pseudocalanus* was similar during both years [21]. The collection of Pacific copepods *E*. *bungii* and *M*. *pacifica* during August and September further illustrated the transport of warm Pacific waters into the Chukchi Sea. Most meroplankton collected during summer and autumn 2018 consisted of barnacle larvae, in agreement with observations of barnacle larvae contributing to the largest meroplankton biomass from 2008 to 2010 in the northeastern Chukchi Sea [62].

Despite higher water temperatures during summer and autumn 2018, coinciding with record high surface seawater temperatures in the Pacific Arctic [20], water temperatures rapidly decreased and sea ice quickly formed in November at DBO4 and in early December at DBO2 and DBO3, leading to the prolonged presence of cold water at all sites during winter 2018–2019 and to a later sea ice breakup at DBO2 in 2019. Cells of the ice-associated diatoms *Fragilariopsis* spp. were first collected simultaneously to sea ice breakup at the end of April at DBO2 and DBO3 and during mid-May at DBO4, indicative of the latitudinal delay in sea ice breakup. Peak diatom fluxes were consistently observed approximately three weeks after sea ice breakup, resulting in a 2-week delay between the diatom bloom in the northern Bering Sea (DBO2) and in the northern Chukchi Sea (DBO4). Regardless of the delay in the onset of the bloom, the composition of the diatom fluxes was strikingly similar at the three sites during 2019, with *Fragilariopsis* spp., *F*. *arctica*, *Navicula* spp., *Pseudo-nitzschia/Nitzschia* spp., *Thalassiosira* spp., and *Chaetoceros* spp. composing most of the fluxes. These observations indicate that algal production was likely back to normal in the northern Bering Sea.

Whereas Pacific copepods were not collected in 2019 despite the enhanced transport of Pacific waters observed until September 2019 across Bering Strait [7], the sustained inflow of warmer waters resulted in the near absence of *Calanus* at DBO2 and DBO3 during June and July 2019. While the majority of *Calanus* copepods present in the Chukchi Sea are believed to originate from the Bering Sea [38], their increasing abundance at DBO4 in July 2019 suggested an alternate source of *Calanus* on the northern Chukchi shelf during summer 2019 [63]. No clear peaks in copepod abundance were observed during June or July 2019, consistent with high seasonal and interannual variability in the Pacific Arctic region [62]. By contrast, peaks in nauplii abundance recorded during June 2018 and 2019 at DBO2 and DBO3 revealed a steady timing in copepod reproduction despite variations in the timing of sea ice breakup. Finally, the concurring absence of diatoms and copepods of Pacific origin at the DBO sites during 2019 suggested that less productive conditions further south may have limited their advection into the region that year.

### Less ice led to generally lower annual export fluxes

A comparison of annual fluxes obtained for each deployment cycle highlights the large-scale impact of an early sea ice breakup on export in the Pacific Arctic region. In the northern Bering Sea, lower annual TPM, POC, chl *a*, and diatom fluxes during the June 2017-June 2018 cycle than during the June 2018-July 2019 cycle, despite the export of a large pelagic bloom at the end of May and early June 2018, indicated that elevated fluxes were sustained over a longer period when ice breakup occurred a few weeks later during 2019. It is reasonable to assume that annual fluxes would have been higher for the 2017–2018 cycle without the interruption in sampling during June 2018. However, it is unlikely that these annual fluxes would have been higher than during 2018–2019 as decreasing near-bottom chl *a* concentrations recorded at a close-by mooring indicated a declining bloom during June 2018 [17]. Lower annual chl *a* fluxes during 2017–2018 may have also partly arisen from the dominance of the generally small-sized *Chaetoceros* cells with lower chl *a* content during the 2018 spring bloom. The large abundance of copepodites and nauplii during summer 2018, together with the larger annual FPC flux during 2018–2019, suggest that the massive pelagic bloom enhanced secondary production and grazing pressure in the northern Bering Sea, supporting the hypothesis of a transition to a pelagic-dominated ecosystem under warmer conditions [64]. The dominance of small-sized diatoms and copepods under higher water temperatures and reduced ice cover in the northern Bering Sea concurs with observations in the Atlantic Arctic sector where a shift in the composition of the phytoplankton and zooplankton communities was observed during a period of anomalously warm Atlantic Water inflow and absence of sea ice cover in the eastern Fram Strait [65]. These observations therefore suggest a similar impact of warmer conditions on the Arctic marine ecosystems influenced by the inflow of Pacific and Atlantic waters. Lastly, lower annual TPM and POC fluxes during 2017–2018 along with similar autumn and winter peaks in daily TPM and POC fluxes during both cycles suggest that the intensity and frequency of resuspension events did not significantly increase during the period of unprecedented sea ice loss during 2017–2018 in the northern Bering Sea.

In the southern Chukchi Sea, annual fluxes were also lower during the 2017–2018 cycle than during the 2018–2019 cycle, except for a slightly higher annual FPC flux. The lower chl *a* and diatom fluxes collected at DBO3 during 2017–2018, despite sea ice breakup actually occurring later during 2018 than during 2019, highlighted the influence of advected waters from the northern Bering Sea. Indeed, a shorter period of lower diatom fluxes at DBO3 during May 2018 possibly resulted from a reduced inflow of nutrients into the southern Chukchi Sea following the massive pelagic bloom that occurred during the prior weeks in the northern Bering Sea. Whereas higher annual FPC flux during 2017–2018 may be the result of larger fecal pellets produced by larger copepods during summer 2017, the highest copepod abundance during summer 2018 also contributed to relatively high FPC fluxes at DBO3 during the 2018–2019 cycle, further supporting a transition to a pelagic-dominated ecosystem under warmer conditions [64]. Although annual TPM and POC fluxes were approximately half as high during the 2017–2018 cycle as during 2018–2019, mostly due to the enhanced daily TPM and POC fluxes sustained during the bloom period from May to July 2019, the high springtime POC fluxes recorded during both years when compared with POC fluxes from around the world [19] indicate a persistently strong biological pump in the region due to the highly productive and shallow nature of the shelves.

By contrast to the DBO2 site where sea ice conditions were heavier during 2018–2019 than during 2017–2018, sea ice breakup at the DBO4 site took place more than two months earlier during 2019 than when the same site was previously sampled during 2016 [21]. Considering that the day of sea ice breakup on the Chukchi shelf ranged from mid-May to mid-August between 2010 and 2017 [66], sea ice breakup occurred relatively early during mid-May 2019 at DBO4. This early sea ice breakup led to lower annual chl *a* and intact diatom fluxes during 2018–2019 than under longer lasting sea ice cover during 2015–2016 [21]. While Waga and Hirawake [67] reported an increasing occurrence of fall blooms using satellite remote-sensing data from 2003 to 2017 for the Chukchi Sea, the absence of large diatom fluxes during autumn 2018 in contrast to autumn 2015 partly explains the lower annual chl *a* and diatom fluxes recorded during 2018–2019. However, the lower annual chl *a* and diatom fluxes observed near Hanna Shoal also resulted from the short-lived and smaller peak in diatom fluxes recorded after sea ice melt during spring 2019, while elevated diatom fluxes were sustained during several weeks in the presence of ice cover during spring and summer 2016 [21]. Therefore, a loss of sea ice resulted in a shorter period of enhanced springtime diatom fluxes during 2019 at DBO4, similar to observations during spring 2018 at DBO2 and DBO3. By contrast, annual TPM and POC fluxes were higher during 2018–2019 than during 2015–2016 at DBO4 [21], principally due to high daily TPM and POC fluxes following sea ice melt during spring and summer 2019. Elevated TPM and POC fluxes at a time of lower chl *a* and diatom fluxes during summer 2019 suggest that enhanced resuspension under reduced ice cover likely contributed to these fluxes.

## Conclusions

Overall, earlier sea ice breakup resulted in shorter periods of elevated chl *a* and diatom fluxes following sea ice melt at the three DBO sites on the shallow Pacific Arctic shelves. While POC fluxes represent diverse sources of particles, such as material released from the ice, resuspension events, and lateral advection, chl *a* and intact diatom fluxes more reliably reflect the impact of reduced sea ice conditions on the biological pump. Chl *a* and diatom fluxes obtained during the two-year sediment trap time-series therefore suggest that the recent loss of sea ice and ensuing weaker stratification reduced the strength of the biological pump in the Pacific Arctic region. In addition, the sustained transport of warm Pacific waters [7] led to the low abundance or absence of *Calanus* copepods at the three DBO sites during the study period. The abundance of smaller *Pseudocalanus* copepods instead either increased or decreased FPC fluxes and the efficiency of the biological pump at the DBO sites, highlighting the need for additional export flux measurements to determine long-term trends. As warming of the Arctic and sub-Arctic continues, major ecosystem changes are expected to occur more frequently in the Pacific Arctic region [14]. In this context, sediment trap time-series provided valuable, nearly uninterrupted measurements of biological parameters at a high temporal resolution to complement the other repeated observations made as part of the DBO. Routine sediment trap deployments would help determine if the changes observed under enhanced transport of warmer Pacific waters and shorter ice-covered duration represent an anomalous period or a new normal for the Pacific Arctic marine ecosystem.
